# What Medical Journal Editing Means to Me

**DOI:** 10.4103/0973-1229.33004

**Published:** 2008

**Authors:** Harvey Marcovitch

**Affiliations:** * *Associate Editor, BMJ and previously Editor, Archives of Disease in Childhood*

**Keywords:** *Journals, Editors*, *Research Ethics*, *Readers, Medical Journal Editing*, *Impact factor*, *Committee on Publication Ethics (COPE)*, *Budding editors*

## Abstract

Papers in medical journals are often difficult to understand and tedious to read. An editor's first loyalty should be to readers, by prioritising readability over merely producing a repository of data for the scientific community generally.

The web now provides infinite repository space so there is even less excuse for journals to be unreadable. I give examples of how I attempted to improve one journal, despite external pressures and regardless of how it might affect the Impact Factor. As a postscript I outline increasing involvement in promoting honesty and integrity in publishing through the auspices of the Committee on Publication Ethics (COPE).

The invitation to edit *Archives of Disease in Childhood* came pretty well out of the blue. True, I had reviewed papers for the journal and, for a couple of years, had been an associate editor assisting with final decisions on whether to accept individual papers. However, there were others far more senior and more to the point, much more academic.

I had no academic pretensions, having spent 25 years of my working life as a full-time UK National Health Service paediatrician in secondary care hospitals. Decades ago I spent 18 months as a research fellow at London's Institute of Child Health neonatal research unit, but my contribution to the sum of scientific knowledge in neonatology was soon eclipsed by others and had vanished into obscurity (Parker, *et al.*, 1971).

## Why is it Impossible to Understand a Word of this?

Thus, my unexpected elevation to an editorial chair served to highlight a paradox, which provides me with endless delight and fascination. It is simply this: it seems to me that the primary purpose of a scientific journal is to communicate to those who wish or need to know, information that an author wishes or needs to impart. Yet, in the early days of my editorship I recall my wife - a journalist by trade - flicking through the journal and complaining ‘why is it impossible to understand a word of this?’ She was right: most scientific journals are a struggle to read, so, by definition, poor transmitters of information. There are many reasons, one of which might be that by having a private language one can exclude others from the art - part of the ancient priestly function of doctors, now long outmoded. Another is simply to do with the medical profession's addiction to pomposity.

The first scientific journal published in the English language (the French got there first with *Journal des Sçavans*) was *Philosophical Transactions of the Royal Society of London*. In introducing the journal, its publisher declared:

*Philosophical Transactions will use the language of artisan countrymen and merchants rather than that of wits and scholars (Sprat, 1667)*.

This desire to use plain English lasted for more than 200 years. In 1859 a Lancet editorial about the diet of soldiers started:

*Whilst it is a well known fact that English soldiers both require and receive a larger allowance of animal food than do any other troops, yet, on the other hand, it is equally plain that the defective processes of cookery to which such food is subjected render it frequently insufficient to maintain the soldier's strength* [Editorial (Anonymous), 1859].

The sentence may be a little longer than is now fashionable but compare it with an extract from the *BMJ* nearly 150 years later (reference withheld to spare the authors’ blushes):

*Although concern about the impact of current housing policy on public health was shown by a substantial number of directors, the main activity was still allocation of increasing housing need and homelessness. The underlying need is for greater advocacy to produce a healthy housing policy for all and the annual public health report could be used to promote this objective*.

### Or even

*It has been established that the curtailed treatment schedule may offer perceived social and medical advantages for patients, with the additional benefit of it becoming moiré economical*.

I guess the first of these authors was trying not to say something straightforward like:

*Many directors of public health were concerned about how housing policy might affect health but spent most of their time dealing with who should get the few houses available. They could use their annual reports to promote a housing policy that took health into account*.

### And the Second Seems to Mean

*Patients prefer the shorter treatment and it's cheaper*.

(I am grateful to Tim Albert, trainer of medical writers and editors, for these extracts.)

Artisan countrymen don't speak like the original quotations, so what happened and how does it fit in with my perceptions of editorship? Medical writing has been infected with what a distinguished UK editor, Dr Michael O'Donnell, called ‘decorated scientific gothic’ (O'Donnell, 1997). Think of the older buildings of Mumbai University or, for those familiar with London, St Pancras Station. The grandiosity that Victorian architects believed was necessary to declare that a building was important has its echo in much of the language now used by medical scientists. Such is the insistence on using a specially medicalised form of the English language to denote scientific credibility that, at a course for medical editors, a senior editor argued vigorously with me that ‘about’ did not mean the same as ‘approximately’ because the latter was ‘more scientific’.

## Primary Loyalty to Readers Rather than Authors

When I took on my editorial role, my main preoccupation was for my *primary* loyalty to be to my journal's readers rather than to aspirant authors, the demands of the publishers, the requirement of the co-owner the Royal College of Paediatrics and Child Health or even to the scientific record itself. What I felt important was not so much that the journal should simply provide a repository for data but rather that its readers might look forward eagerly to its arrival every month and even go so far as to open the cover page, let alone take off the wrapper (O'Donnell, 2005). Not every editor agrees: there are those who say the purpose of their journal is simply to document the work that is being done in their specialist field. That may be true of small and esoteric specialties where the protagonists mostly know each other and authors and readers are indistinguishable. It may even be true that there are authors who do not mind whether or not their contribution is read - what matters to them is that it is published (Healy, 1976). For, as we know, status, promotion and therefore income, depends for some on the number of times their name appears on a title page multiplied by the impact factor of the journal concerned.

Those who still subscribe to this mid-twentieth century view of medical publishing are dinosaurs that, I hope, the cataclysm of the Internet will render extinct. In the developed world and in much of the developing world, we no longer need paper journals simply as (inefficient) data storage devices. Scientists can store as much as they like in whatever form they wish in cyberspace and those who really need to study their work in depth can do so with ease. But the large majority of medical journal readers, especially those of the general journals, do not need to do so. Sophisticated readership surveys and the sort of straw poll you might undertake amongst your colleagues, will show that an individual reader may ignore much, skim some and read little of the content. As I see it, my editorial task was to grab them by the eyeballs and make sure they do more of the last than the first. My motives were partly altruistic and partly egotistical: altruistic, because I truly believe that having well-informed clinicians, public health doctors and basic scientists is good for patients. Egotistical, because I gain pleasure in hearing acquaintances say how much they enjoyed reading a particular issue, series or editorial.

## From Scarcely Readable to Attractive to Look at

Changing a journal from being scarcely readable to being attractive to look at is not something to be done overnight. The medical readership is conservative and suspicious of change. Editors, unless they run prestigious and profitable journals whose impact factor they have raised to the stratosphere, are always vulnerable to the beliefs or prejudices of their editorial board or the institution that employs them. Even the exalted are vulnerable as evidenced by the sacking or resignation of previous editors of the *Journal of the American Medical Association*, the *New England Journal of Medicine* and the *Canadian Medical Association Journal*. (Bologna, *et al*., 1999; Garceau, *et al*,, 1999; Spurgeon, 2003; Godlee, 2006; Van Der Weyden, 2006; Singh and Singh, 2006). Experience had told me that what readers of my journal appreciated most were reviews, editorials, controversies, educational pieces and fillers and what they read least often were original scientific papers. Now that we can count hits on journals’ electronic pages, this assumption has proved to be reliable.

That does not mean that editors should consign science to the dustbin. There is a less immediate but nonetheless important role for specialist journals in encouraging research and clinical observation and adding the occasional brick to the wall of knowledge. What it has meant to me is that I had to find ways of adding value to my journal in an analogous way to how chefs add value to ingredients when they produce gourmet meals. Gradually, I introduced or strengthened pre-existing added value in terms of a cover which invited the reader to turn over to the next page; an introductory page highlighting those papers to which I wanted to draw readers’ attention; feature items by gifted medical writers to break up the seemingly ceaseless torrent of original papers; illustrations and filler articles; abstracted relevant material from other journals; innovative educational items - such as ‘quick and dirty’ evidence based searches that could be performed on a ward round and provide a chance for publication by junior clinicians - and many other examples, some more successful than others (Marcovitch, 2002; Phillips, 2001).

On taking early retirement from the National Health Service to work for the *BMJ* Publishing Group as a free-lance editor and the General Medical Council as a member and chairman of panels, which decide upon doctors’ fitness to practice, I was caught by a Royal College rule that editors must be practising researchers or clinicians. My tenure was over after nearly a decade and I handed over to my excellent successor, Professor Howard Bauchner from Harvard Medical School - who is both clinician and researcher. My hope was that I had handed over a journal in better shape than it had been when I started. I hope that I succeeded, although if one takes impact factor as a major criterion, then I failed, as this remained static. I take solace in the fact that I had never paid much attention to this spurious statistic. Undoubtedly the higher impact factors of our American competitors, let alone that of the big international general journals, resulted in papers of value to our readers finding their way elsewhere. This might be a blow to one's pride but, keeping authors’ requirements at the forefront, this corruption of communication flow from writer to reader could be obviated by publishing brief paraphrases of those papers I would have liked to come in my direction in the first place.

## More Like *Cosmopolitan* than Like *Brain*

Dr Richard Smith, previous editor of the *BMJ*, once said that he was awaiting the day that his journal looked more like *Cosmopolitan* (a lifestyle magazine for fashionable young women) than *Brain* (regarded as the epitome of tradition) (Smith, 2002; Compston and Newsom, 2002). His successor, Dr Fiona Godlee, has not gone that far. But the 2007 version of the *BMJ* recognises that the primary scientific publication is actually bmj.com, while the paper journal is there not just to inform but also to entertain and provoke (Godlee, 2007). As an associate editor I play a very minor role in its production; but I am proud to be associated with what I regard as a journal that means a lot to me as a medical journalist.

Any editor will tell you that with the present day electronic copy flow structures, most papers move smoothly, albeit not always rapidly, through the system. Every now and again, however, one comes up against a problem. A paper may detail experiments, which appear unethical or for which there may not have been properly informed patient consent. A reviewer may allege an attempt at redundant or duplicate publication. An editor may discover plagiarism of another's work. Worst of all there may be suspicion of dishonest manipulation of data. The more one looks for such misconduct the more one discovers them and every case takes up a lot of time and emotional energy.

When I left my editor's chair, I hoped that, whatever I had done or not done, I had nonetheless protected the scientific record against corruption and dishonesty. My interest in the subject and my outrage at some of the behaviour I encountered has led to my taking up a task as Chairman of the Committee on Publication Ethics (COPE) and to joining the board of the UK Panel on Research Integrity (COPE Flowcharts, 2007).

So whatever else being an editor meant to me, it has opened up new doors at a time of life when many of my colleagues are firmly closing theirs. What's more, as an editor, I could do something I could rarely do as a doctor - say ‘No’.

## Finally, Here's Something for New or Budding Editors

If there are any new or budding editors reading this, here is what you could do to make the biomedical publishing world a happier place. Don't regard your reviewers as editors. Their opinions may help you come to a decision, but just because they would like to see something published it does not necessarily mean it is right for your journal. The easiest way to decide what is right for your journal is to make sure you have a mission statement: ideally this has one sentence, but if that is asking too much I will allow you two, provided each includes a verb and tells you, your authors and your readers what you are there for. For example, ‘We aim to publish original research and reviews about the inguinal ligament which will help our readers better manage its problems.’ If a paper does not satisfy the mission, no matter how careful the methodology or precise the statistics, suggest it goes somewhere else. Keep an eye on your competitors and if you can't beat them, join them by adopting techniques that look successful in improving the flow of communication from your authors to your readers. Be tough on papers you intend to accept to make sure they hit the spot but be kind with your rejection letters. Finally, never get downhearted by criticism or lack of recognition from your peers. In general the only people who love editors are their wives, husbands, children and parents.

### Take Home Message

Biomedical journal editors control the final gateway to publication. So they must protect the integrity of the scientific record by being knowledgeable (or knowing whom to ask), fair and frank. They are not there just to curate a database but rather to help get their authors’ messages across to readers. That means it is not enough to be a skilled and experienced researcher or clinician. Journalism, too, has its knowledge base and best evidence. By tapping into the skills of this non-scientific specialty, editors will reap rewards for themselves, their readers and the scientific community at large.

## About the Author

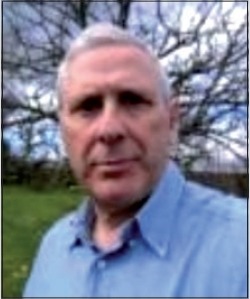



Harvey Marcovitch was a consultant paediatrician from 1977-2001, latterly at the Oxford Radcliffe Hospitals NHS Trust. From 1994-2002 he was editor of Archives of Disease in Childhood and is currently syndication editor for the BMJ Publishing Group and Associate Editor of the BMJ. He is editor of Black's Medical Dictionary and a chapter author of ‘How to Write a Paper’ (ed Hall G, 3^rd^ edn, BMJ Books, 2003) and Medicine for Lawyers (ed. Palmer R, Wetherill D, RSM Press, 2005). In 2006 he became chairman of the Committee on Publication Ethics (COPE) an organisation with over 300 editor and publisher members from Europe, Asia and elsewhere. He is on the board of the UK Panel for Research Integrity and a member of the editorial policy committee of the Council of Science Editors (US). He also chairs fitness to practice panels of the UK General Medical Council.
